# How to reload and upgrade digital health to serve the healthcare needs of Nigerians

**DOI:** 10.3389/fdgth.2023.1225092

**Published:** 2024-01-18

**Authors:** Nirmal Ravi, Christy Thomas, Juliet Odogwu

**Affiliations:** ^1^ EHA Clinics, Kano, Nigeria; ^2^ eHealth Africa, Kano, Nigeria

**Keywords:** digital health, Nigeria, artificial intelligence, digital infrastructure, primary healthcare, Africa, interoperability

## Introduction

### Digital health

The term “digital health” is rooted in eHealth, described at the turn of this century as “the use of information and communications technology in support of health and health-related fields.” (1) Early iterations of eHealth and mHealth technologies included electronic medical records and their analyses, SMS patient notifications and reminders, automated reports, and clinical decision support systems. Today, the scope of “digital health” encompasses a panoply of products and services, including virtual telemedicine consultations, online pharmacies, at-home laboratory test services, wearables, and digital therapeutics. Unlike with earlier waves of digitisation that rolled through the telecommunications and banking sectors though, many developing countries, including Nigeria, have yet to adopt and reap the benefits of these technologies and services. We opine that this neglect is leading to needless inconvenience and suffering for many who are looking for cheap and effective access to healthcare services.

### The importance of digital health

Health systems globally face tremendous problems as they aim to deliver better value healthcare amid rising chronic disease, ageing populations, climate change, and a gloomy economic forecast. Digital health tools and services are frequently hailed as part of the answer, delivering low-cost, patient-centred solutions. This includes internet-connected devices that let doctors remotely monitor their patients, mobile apps to raise awareness about diseases, telemedicine consultations between patients and clinical providers, and electronic health records that illustrate a patient's medical history. After a virtual consultation, patients could order an at-home laboratory test to help make a diagnosis, and have treatments delivered from an online pharmacy. Artificial intelligence (AI) algorithms can find hidden patterns in data, interact with natural language, and support the automation of interactions. Healthcare professional decision-making can be augmented with clinical decision-support tools powered by AI capabilities. Digital therapeutics—digiceutical in the jargon—is a particular class of software as a medical device (SaMD) that are approved by stringent regulatory authorities such as the US Food and Drug Administration, and are prescribed by a doctor to provide real-time guidance to patients (2).

In May 2018, a historic resolution on digital health that was submitted by India and 14 other WHO Member States was adopted at the 71st World Health Assembly (3). WHO highlights that digital health may help achieve the Sustainable Development Goals by enabling universal access to high-quality health and wellness services for all people worldwide. It put forth a Global Strategy on Digital Health 2020–2025, with several countries having already achieved key milestones (4).

### Healthcare in Nigeria

Nigeria, the largest economy and most populous country in Africa, faces a daunting array of healthcare challenges. In terms of health indicators, Nigeria has some of the highest rates of maternal and child mortality in the world, as reported by the recent Lancet Nigeria Commission (5). Communicable and non-communicable diseases both contribute significantly to the overall disease burden. The country also grapples with high levels of poverty, inequalities in the distribution of services due to a lack of healthcare facilities in rural areas, a lack of material and human resources, poor infrastructure, low government investment in health system financing, broken supply chains, fragmented markets, and lack of quality while providing healthcare services. Over 74.7% of the population in Nigeria is still forced to pay for healthcare out of their own pockets (6).

### Current digital health scenario in Nigeria

Beginning in late 2014 and continuing into the first half of 2015, the Nigerian Federal Ministry of Health (FMOH) and Federal Ministry of Communication Technology (FMCT) coordinated the multi-sectoral and stakeholder development of the National Health Information and Communication Technology (Health ICT) Strategic Framework, which was expected to run between 2015 and 2020 (7).

The National Health ICT strategic framework started with a clear set of visions that were focused on Universal Health Coverage in Nigeria. The primary vision of the framework was that “By 2020, health ICT will help enable and deliver universal health coverage in Nigeria.” The vision sought to improve coverage of health services through the effective use of Civil Registration and Vital Statistics (CRVS), National Identity Management System (NIMS), Human Resource Management Information Systems (HRIS), National Health Management Information System (NHMIS), and Logistic Management Information System (LMIS) for tracking demand and supply of health services and commodities. All of these were to be backed by a national Health Information Exchange (HIE) and implemented with the required governance, funding, infrastructure, training, and policies.

The Nigeria ICT-enabling environment for health is transitioning from experimentation and early adaptation to development and building. A recent review of the landscape and inventory of ICTs for health within Nigeria demonstrated that more than one hundred distinct ICT initiatives were implemented throughout the country to improve an array of health system functions (8). Although there are barriers and challenges in many of the core component areas, it is most important to focus on sustainability and filling the gaps.

The sustainability of digital health interventions holds paramount importance in upholding the standards of healthcare, particularly within low and middle-income nations (9).

However, healthcare professionals in Nigeria are optimistic about the ability of digital health to improve healthcare delivery and accessibility. They highlight the convenience of telemedicine for distant patients, electronic health records for streamlined patient information management, and AI-assisted diagnosis for enhanced clinical decision-making (10). However, healthcare professionals encounter challenges in the adoption and implementation of digital health solutions attributable to insufficient training in novel digital tools, issues pertaining to internet connectivity, and suboptimal technical support infrastructure (11). They believe that leveraging digital technology serves to bolster the cost-efficiency of healthcare provision and broaden the accessibility of services to geographically isolated regions of Nigeria (12).

A comprehensive understanding of the role of digital health in Nigeria is aided by empirical research capturing these diverse perspectives. It informs strategies that cater to the specific needs of healthcare professionals, fostering an environment in which digital health can genuinely improve healthcare outcomes.

## Discussion

### Barriers and challenges to digital health practice in Nigeria

Despite making a strong start, Nigeria's efforts to bring healthcare into the digital age have recently foundered. Numerous obstacles, from a shortage of trained healthcare personnel to IT infrastructure persist, making implementing effective digital health solutions in Nigeria challenging. Nigeria boasts one of Africa's largest human resources for health (HRH) stocks, but the number of nurses, midwives, and physicians remains insufficient to provide essential health services effectively, with only 1.95 per 1,000 population (13). Existing infrastructure and resources are inadequately distributed, with most services concentrated in urban tertiary health centres. Many hospitals and primary health care clinics on the state level lack trained personnel and equipment, and many others struggle to function due to frequent power outages that last for extended periods. Robust deployment of digital health solutions and services can offer a way to leapfrog some of these hurdles, the way digital solutions have transformed banking in Nigeria. Here we identify several areas that need more attention to advance digital health in Nigeria.

## Recommendations

### Digital health in Nigeria: a way forward

#### Leadership, strategy and governance

Nigeria is a federal country with state and local governments responsible for delivering the majority of primary and secondary healthcare. This has led to the weak uptake of a centrally promulgated national health ICT strategic framework. Competing federal, state, and local government programmes and priorities further impede the establishment of a coordinated cross-ministerial health ICT strategy, contributing to a low level of transparency, segmentation, market risk, and high prices. Developing an inclusive national strategy is essential to building a sustainable initiative that can address a country's most critical health requirements. This strategy must include future investments in technologies, and provide a policy framework and a unified set of targets. We especially like the practical guidelines in the WHO “Digital Implementation Investment Guide (DIIG): Integrating Digital Interventions into Health Programmes.” (8) We recommend the establishment of a national digital health advisory committee, including private sector providers, similar to Singapore's approach, to bring all stakeholders and drive progress.

#### Human resources

To ensure that healthcare providers are equipped to use digital health effectively, professional bodies like the Medical and Dental Council of Nigeria can make it mandatory for all providers to undergo training in giving telemedicine consultations. High level of understanding and acceptance of telemedicine and digital health among healthcare professionals is crucial for its successful implementation because these concepts are still relatively new (10). In these early days of digital health and telemedicine, Nigeria can adapt the detailed Telemedicine Practice Guidelines published by India that walk providers through a flowchart, ensuring the appropriateness of care provided at the same standard of care delivered in person (14). When setting such standards, policymakers must beware of setting the standards higher than that required by in-person care. For instance, Nigerian pharmacy regulations prohibit the prescription of antibiotics through telemedicine. This will only perversely increase inappropriate antibiotic use by driving patients to bypass telemedicine consultations and buy medicines directly from pharmacies without oversight. Similarly, policymakers must not shy away from formulating supportive policies on AI-assisted diagnosis. As long as a registered clinical provider is “in the loop,” AI algorithms, when appropriately developed, validated and monitored, have great potential to improve healthcare delivery. Training and equipping community health workers with clinical decision support software has the potential to backstop the worsening shortage of physicians, especially in rural areas.

#### Health system financing and private investment

Financing is a fundamental prerequisite for the sustainability of digital initiatives. Over the years, funding schemes have been introduced to address shortages in funding- NHIS and the National Health Bill. But, in the private sector, there is almost no additional investment in primary care, as the return on investment is much higher from tertiary care and specialised hospitals performing niche services such as cardiac care, joint replacements, eye care, etc.

Many digital health interventions in Nigeria such as the hotline for TB, start as pilot programs supported by donors, left inoperative once donor priorities change. Private investment has been entering many digital health services in Nigeria, such as women's health and sexual health, when there is a clear business case and path to profit. These services must now pair with quality-assured brick and mortar healthcare service providers to benefit patients and keep the momentum for further investment and better health outcomes.

#### Infrastructure

In building sustainable health systems, both old-world and new-world infrastructure are essential. The telecommunications sector in Nigeria has undergone significant growth in mobile cellular subscriptions, which currently stands at 91 per 100 people (15). The parameters of this connectivity, including its geographic coverage, quality, speed, and cost will play crucial roles in determining the uptake of digital health. Teleconsultation between a doctor and patient is more reliable and effective when the communication happens over a video call than on a voice call. However, this can aggravate the digital divide across rural and urban areas, as the majority of rural communities in Nigeria either completely lack mobile internet or have them at speeds of 2G. It is imperative that we don't leave behind those who don't have or don't know how to use smart phones. Call centres staffed by specially trained frontline healthcare workers equipped with clinical decision support systems can screen and triage patients to the right solution provider. With interoperable systems, they can check the availability of diagnostic tests and medicines in pharmacies close to the patient, and direct them to the appropriate locations.

#### Data protection and interoperability

Only with interoperable health data systems can data be integrated and distributed among diverse actors, which plays a significant role in sustainability. In Nigeria, there is currently no national authority accountable for endorsing particular health informatics standards. As artificial intelligence transforms our everyday interaction with data, large annotated health datasets are necessary for training machine learning algorithms. This will certainly not be possible in Nigeria if healthcare providers and IT solution providers do not use standard terminology and practices to capture and store data. Regulatory bodies can enable this by defining or recommending existing standards for processing healthcare data in Nigeria.

Public faith in data privacy can improve the willingness to freely use digital health services offered by healthcare providers (16). Providers of digital health must take data privacy very seriously as a breach can roll back significant progress. Just as fintech has been able to address customers’ concerns through robust security architecture and processes, health data can also be secured with proper risk management and mitigation measures. Misguided government policies requiring data storage within national borders can backfire since many machine learning algorithms require cloud storage, and run in specialised data centres. Once there is a critical mass of digital health providers within Nigeria, there will be a natural business case for cloud service providers to open data centres locally.

## Conclusion

Digital health is not a panacea for the healthcare problems of Nigeria; but it must be an indispensable and principal component of the future healthcare system in Nigeria. Millions of people who would otherwise go to the nearest kiosk for whatever is ailing them can benefit from digital health to seek and receive quality healthcare. But, for this to happen, it must be as easy and efficient to use as it is to walk to that kiosk. Regulatory and governing bodies must establish an affirmative environment through meaningful and inclusive consultations with all stakeholders. Capital, both public and private, must flow to their respective service points guided by the common guardrails of patient safety and return-on-investment. Healthcare providers must be comfortable using software tools to help them think beyond the crutches of “malaria and typhoid” when a patient is looking for answers. And by incrementally building on the already existing technology infrastructure, Nigeria can reload and upgrade to a new digital healthcare wave that follows fintech and telecommunications.
